# Phosphatidic Acid (PA) can Displace PPARα/LXRα Binding to The EGFR Promoter Causing its Transrepression in Luminal Cancer Cells

**DOI:** 10.1038/srep15379

**Published:** 2015-10-23

**Authors:** Madhu Mahankali, Terry Farkaly, Shimpi Bedi, Heather A. Hostetler, Julian Gomez-Cambronero

**Affiliations:** 1 Wright State University School of Medicine, Department of Biochemistry and Molecular Biology, Dayton, Ohio 45435, USA.

## Abstract

The expression of the epidermal growth factor receptor (EGFR) is highly regulated in normal cells, whereas some cancer cells have high constitutive levels. Understanding naturally-occurring ways of downregulating EGFR in cancer cells was investigated. Phosphatidic acid (PA) or Nuclear Receptors (NR) PPARα/RXRα/LXRα, enhance EGFR expression, mediated by the promoter region -856(A) to -226(T). Unexpectedly, the combination of NRs and PA caused repression. PA induces a conformational change in the nuclear receptor PPARα (increase of alpha-helices at the expense of decreasing beta-sheets), as evidenced by circular dichroism. This represses the naturally-enhancing capability of PPARα on EGFR transcription. PPARα-overexpressing cells in the presence of PA > 300 nM or the enzyme that produces it, phospholipase D (PLD), downregulate EGFR expression. The reasons are two-fold. First, PA displaces PPARα binding to the EGFR promoter at those concentrations. Second, NR heterodimer-dependent promoter activity is weakened in the presence of PA *in vivo*. Since other genes considered (β-catenin, cyclin D3, PLD2 and ACOX-1) are also downregulated with a PA + PPARα combination, the transrepression appears to be a global phenomenon. Lastly, the reported effect is greater in MCF-7 than in MDA-MB-231 breast cancer cells, which could provide a novel basis for regulating excessive expression of EGFR in luminal cancer cells.

Epidermal growth factor receptor (EGFR) is a transmembrane protein that transmits signals upon binding to ligands such as epidermal growth factor (EGF), transforming growth factor β (TGFβ) and neuregulins^1^,^2^. EGF/EGFR signaling is essential in development via its roles in cell cycle progression, differentiation, cell movement and survival^3^,^4^,^5^. EGF signaling plays an important role in phospholipid metabolism via its ability to regulate phospholipase Cγ (PLCγ), phospholipase D (PLD) and phosphatidylinositol-3-kinase (PI3K). Our lab and others have also shown in previous studies an activation of phospholipase D2 (PLD2) through EGFR in cancer cells^6^,^7^,^8^,^9^,^10^ however, no long-term studies of PA on the receptor have yet been documented.

A direct role of EGFR in the process of tumor invasion and dissemination has been shown^11^. Furthermore, overexpression of EGFR is one of the frequent mechanisms implicated in cancer progression^11^,^12^. It has been suggested that transcription factors that positively regulate EGFR promoter activity are also overexpressed in many cancers^13^,^14^. The EGFR promoter has multiple initiation sites and also has binding sites or response elements for many transcription factors, including Sp1, p53, interferon regulatory region-1, estrogen, vitamin D and retinoic acid^15^,^16^,^17^.

Phosphatidic acid (PA) is formed naturally in the cell due to the catalytic action of PLD on phosphatidylcholine (PC)^18^. Growth factors and other physiological stimuli are known to activate PLD2, which contributes to increasing PA levels in the cell. PLD and PA interact with a wide variety of proteins and have been shown to be involved in cancer metastasis progression. Similarly, PLD inhibitors decrease tumorigenesis in murine models^19^,^20^. Stimulation of cells with growth factors, such as EGFR, induces PLD activity and furthermore EGFR directly interacts with PLD2^6^,^21^,^22^,^23^. In ovarian cancer cells, EGF signaling induces PLD2, which is responsible for the production of lysophosphatidic acid (LPA)^24^.

The Peroxisome Proliferation Activated Receptor (PPAR) family of receptors belongs to the nuclear receptor super family, which are major regulators of fatty acid oxidation. PPARα, a type II nuclear receptor (since its subcellular location is irrelevant to ligand activation), binds to DNA response elements in promoter regions of target genes and acts in a heterodimeric fashion by binding to retinoid X receptor (RXRα) or liver X-receptor (LXRα)^25^. PPARα receptors regulate gene expression both positively and negatively by acting as coactivators or corepressors, respectively^26^. PPARα heterodimers might also cause transrepression of the target genes^26^,^27^,^28^.

PPARα is involved in EGFR phosphorylation and activation^29^,^30^. However, PPARα’s direct effect on EGFR promoter is not known. In the present study, we focused on understanding the regulation of EGFR expression at protein and gene levels by PPARα and PA/PLD2. The data presented here indicate that, while separately, PA and PPARα have a positive effect on augmenting EGFR gene expression, and in combination the result is not additive or synergistic activation but rather, transrepression. We report that upon binding of PA to PPARα significant changes in its secondary structure are observed such that the expression of its target gene (EGFR) is repressed. This is because PA interferes with binding of PPARα to the promoter and because it impedes proper recruitment of co-activators RXRα and LXRα.

## Materials and Methods

### Reagents

Dulbecco’s modified Eagle’s medium (DMEM) was from Mediatech (Manassas, VA); Opti-MEM, Lipofectamine, Plus reagent and Lipofectamine 2000 were from Invitrogen (Carlsbad, CA); Transit2020 transfection reagent was from Mirus (Madison, WI); [^3^H]butanol was from American Radiolabeled Chemicals (St. Louis, MO); [^32^P]γATP was from Perkin-Elmer (Waltham, MA); ECL reagent was from GE Healthcare (Piscataway, NJ); EGF was from Peprotech (Rocky Hill, NJ). The plasmids used in this experiment were as follows (all human ORFs): pcDNA3.1-mycPLD2-WT, pcDNA3.1-mycPLD2-K758R, pEGFP-Spo20PABD-WT, pSG5PPARα, pSG5-RXRα and pSG5-LXRα.

### Cells and cell culture

MDA-MB-231 and COS7 cells were obtained from the American Type Culture Collection. MCF-7 cells were a gift from Dr. Steven Berberich (Wright State University). COS7, MCF-7 and MDA-MB-231 cells were cultured in DMEM supplemented with 10% (v/v) fetal bovine serum (FBS).

### Lipid preparation

Lipids from Avanti Polar Lipids (Alabaster, AL) were prepared from powder in “stock buffer”: PBS/0.5%BSA (50 mg de-lipidated BSA per 10 ml of 1x PBS) pH = 7.2, with a final concentration of lipids of 1 mM. This solution was sonicated on ice (at medium setting): once for 4 secs; kept on ice for 4 secs, and this cycle was repeated twice more and extruded (Avanti Polar Lipids). Lipids were kept on ice, overlaid with N_2_ in the tubes, tightly caped, and stored at 4 °C, protected from light in a desiccator. An intermediate dilution (10 μM) was prepared on the day of the experiment in HBSS + HEPES (0.24 g HEPES/100-ml bottle of HBSS), 0.5% BSA, pH to 7.35. Lipids were added (drop-wise) to the cells (30 μl per 1 ml of cells) for a final concentration of 300 nM unless otherwise indicated. Except were noted in the text, most experiments were performed with the phospholipid 1,2,-dioleyl phosphatidic acid (DOPA).

### Gene overexpression and silencing

The protocol for overexpression involved transfection of PLD2-WT, PLD2-K758R, PPARα, LXRα, and RXRα plasmid DNAs into COS-7, MDA-MB-231 or MCF-7 cells using Transit-2020 (Mirus, Madison, WI). Appropriate amounts of DNA were mixed with the transfection reagent in Opti-MEM medium (Invitrogen) in sterile glass test tubes and incubated for 15–30 min at room temperature. Transfection mixture was added to cells in complete media and incubated for 48 h at 37 °C. The cDNA encoding full-length hPPARα, hRXRα and hLXRα cloned into the mammalian expression plasmid pSG5^25^ were a kind gift of Dr. S. Dean Rider, Jr. (Wright State University). Two different sets of silencer siRNAs were used to silence PPARα, from LifeTechnologies (Carlsbad, CA): NM_001001928.2 (targeting exon) and NM_005036.4 (targeting exon 5). The silencing effect was highly effective for each siRNA and varied only between + 15% SEM and as such, data were averaged within each target. To initiate the transfection, siRNA was mixed with Opti-MEM and incubated at room temperature for 5 min, then added to the cells. Transit2020 transfection reagent was used for all other transfections (4 days).

### Cell treatments

For PA, cells that were mock-transfected or transfected with nuclear receptors were treated with increasing concentrations (as indicated in the appropriate figure) of 1,2,-dioleyl phosphatidic acid (DOPA) for 4 to 5 h. Our laboratory has shown previously that this form of PA is cell soluble^31^. Post-treatment cells were harvested and subjected to immunoblot analysis.

### Real Time (Quantitative) RT-PCR (qRT-PCR)

Total RNA was isolated from COS-7, MDA-MB-231 and MCF-7 cells with the RNeasy minikit (Qiagen, Valencia, CA). RNA concentrations were determined and equal amounts of RNA (0.5 μg) were used analyses. Reverse transcription coupled to qPCR was performed following published technical details^32^.

### *In Vitro* PLD Assay

PLD activity (transphosphatidylation) in cell sonicates was measured in liposomes of short chain PC, 1,2-dioctanoyl-sn-glycero-3-phosphocholine (PC8), and [^3^H]-butanol. Approximately, 50 μg of cell sonicates were added to microcentrifuge Eppendorf tubes containing the following assay mix (120 μl final volume): 3.5 mM PC8 phospholipid, 1 mM PIP_2_, 75 mM HEPES, pH 7.9, and 2.3 μCi (4 mM) of [^3^H]butanol. The mixture was incubated for 20 min at 30 °C, and the reaction was stopped by adding 300 μl of ice-cold chloroform/methanol (1:2) and 70 μl of 1% perchloric acid. Lipids were extracted and dried for thin layer chromatography (TLC). TLC lanes that migrated as authentic PBut were scraped, dissolved in 3 ml of Scintiverse II scintillation mixture and counted. Background counts (boiled samples) were subtracted from experimental samples. For some experiments, liposomes were made with 1,2-dimirystoyl-sn-glycero-3-phosphocholine or 1,2-diarachidonoyl-sn-glycero-3-phosphocholine.

### Purification of PLD2

Large-scale overexpression of PLD2, as originally detailed by Gomez-Cambronero *et al*.^33^, was set up from baculovirus, starting from a virulent Bac-C1-myc-PLD2-WT recombinant virus used to infect Sf21 insect cells. Lysates from Sf21 cells (2 × 10^6^ cells/ml) in a spinner of Complete Grace’s Insect Cell Culture Media were used to bind 6xHN-tagged proteins in TALON resin (Clontech) according to the manufacturer’s instructions. Washing buffer was 50 mM sodium phosphate, pH7.0–7.5, 5 mM imidazole, 300 mM NaCl, and elution buffer was 50 mM sodium phosphate, pH 7.0–7.5, 500 mM imidazole, 300 mM NaCl. Optical density at 280 nm was read from eluates of columns. Fractions were then dialyzed (5 mM HEPES, pH 7.8, 50 mM NaCl, 1 mM DTT, 5% glycerol) for 2 h and then frozen at −70 °C for long-term storage. Aliquots were used for SDS-PAGE and for immunoblots that showed the prevalence of a protein at ~110 kDa for PLD2. Yields were ~0.1 μg/μl and we used 50 μl (~5 μg) for assaying lipase activity.

### Protein-lipid binding assays

#### (a) Protein-lipid binding to PVDF membranes

This method for preparing and detecting protein-lipid binding has previously been described^34^. Briefly, the following list lipids were spotted on to the PVDF membrane: 1,2-dioleoyl-sn-glycero-3-phosphate (DOPA), 1,2-diarachidinooyl-sn-glycero-3-phosphate (AraPA), 1,2-diMirystoyl-sn-glycero-3-phosphate (DMPA), 1-oleoyl, 2-hydroxy-sn-glycero-3-phosphate (LysoPA), 1,2-dioleoyl-sn-glycero-3-phosphocholine (DOPC) and 1,2-diarachidonoyl-sn-glycero-3-phosphocholine (AraPC) lipids from Avanti Polar Lipids (Alabaster, AL) were spotted onto a PVDF membrane. Alternatively, readymade lipid snoopers (Avanti, Alabaster, AL) were obtained. All the lipids were dissolved in a 2.0:1.0:0.8 ratio solution of MeOH: CHCl_3_:H_2_O and 2 μg lipids were spotted. The membrane was blocked overnight with a 3% fatty acid-free BSA solution. On the following day, membranes were incubated with recombinant PPARα (10 nM) for 1 h, washed extensively with TBS-T and incubated overnight with anti-PPARα antibody. Next day, the membranes were washed and incubated with secondary antibody and the blots were analyzed by chemiluminescence.

#### (b) Protein-lipid binding by quenching of intrinsic aromatic amino acid fluorescence

Prior to Circular Dichroism, optimal binding of hPPARα to PA and other lipids was determined by quenching of intrinsic aromatic amino acid fluorescence, as previously described^35^,^36^. Briefly, 100 nM of hPPARα was titrated against increasing concentrations of lipid (2.5 nM to 1000 nM) in PBS, pH7.5. Emission spectra were obtained at 24 °C upon excitation at 280 nm with a PC1photon counting spectrofluorometer (ISS Inc., Champaign, IL).

### Purification of recombinant human PPARα protein, Circular Dichroism and analysis of secondary structures

Full-length hPPARα protein was used for the binding assays and circular dichroism experiments. The expression and purification of hPPARα has been described^35^. To determine the changes in the secondary structure of hPPARα as a result of binding of PA, circular dichroic spectra of hPPARα (20 mM Tris,pH 8.0, 150 mM NaCl, 10% glycerol) were taken in the presence or absence of lipid with a J-815 spectropolarimeter (Jasco Inc., Easton, MD) as described for hPPARα with fatty acids^35^. Spectra were recorded from 260 to 187 nm with a bandwidth of 2.0 nm, sensitivity of 10 millidegrees, scan rate of 50 nm/min and a time constant of 1 s. For secondary structure analysis, ten scans for each replicate were averaged for percent compositions of α–helices, β -strands, turns, and unordered structures with the CONTIN/LL program of the software package CDpro^35^.

### Transactivation assays

Luciferase reporter assays were performed by using Secrete-pair Dual Luminiscence Assay Kit (Genecopoeia, Rockville, MD). Cells were transfected with pEZX-PG04 EGFR promoter vector and/or PLD2-WT and/or PPARα, RXRα and LXRα plasmid DNAs. Cells were treated with 3 nM EGF 24 h post-transfection for 24 h and assays were performed as per manufacturer’s instructions. Briefly, culture media was collected after 24 h of EGF treatment. Gaussia Luciferase (GLuc) and secreted alkaline phosphatase (SEAP) activities were analyzed simultaneously using the same samples, which allowed normalization of luciferase activities. Normalized luciferase activities were then compared across all samples.

### *In vitro* PPARα binding to dsDNA promoter

PPARα binding to EGFR promoter was performed by an exonuclease-mediated enzyme-linked immunosorbent assay (ELISA)-like assay (EMEA) according to the method described in^9^, and in the present study tailored for purified recombinant PPARα. We chose a putative EGFR promoter sequence [-847(T) to -801(T)] that bears the consensus Response Element (RE), A/G G/A GT C/G A/G, between -840(A) and -834(A). The oligo sequences were: Sense 5′-TTCCAAG**AGCTTCA**CTTTTGCGAAGTAATGTGCTTCACACATTGGCT(T)_14_-NH2-3′; antisense 3′-AAGGTTC**TCGAAGT**GAAAACGCTTCAT**D**ACACGAAG**D**GTGTAACCGA-5′. In bold is the putative binding site for PPARα; in bold and also underlined are the two digoxigenin (**D**) labeled nucleotides. Taking advantage of the (T)_14_ linker, the sense oligo was immobilized inititially to an N-oxysuccinimide ester-coated plate at a concentration of 200 pmol in a 100 μl volume per each well in oligonucleotide binding buffer (50 mM Na_3_PO_4_, pH 8.5, 1 mM EDTA) and washed extensively. The antisense oligo was added and a dsDNA was formed as in^37^. Plate-bound DNA was incubated with 30 ng/well PPARα for 20 min at 37 °C. Then the plate was treated with exonuclease-III for 20 min at 30 °C to eliminate the fraction of probe not bound to PPARα. Exonuclease digestion buffer was 60 mM Tris-HCl, 0.6 mM MgCl_2_, pH 8.0. Protected PPARα-DIG-labeled DNA was detected with enzyme-linked immunoassays for anti- digoxigenin protein conjugates and visualized by chemiluminescence. Negative controls had 30 ng/well BSA instead of PPARα.

### Statistical Analysis

Data are presented as mean + SEM. The difference between means was assessed by the Single Factor Analysis of Variance (ANOVA) test. Probability of p < 0.05 indicated a significant difference.

## Results

### PPARα has a positive effect on EGFR expression

**Figure 1:**
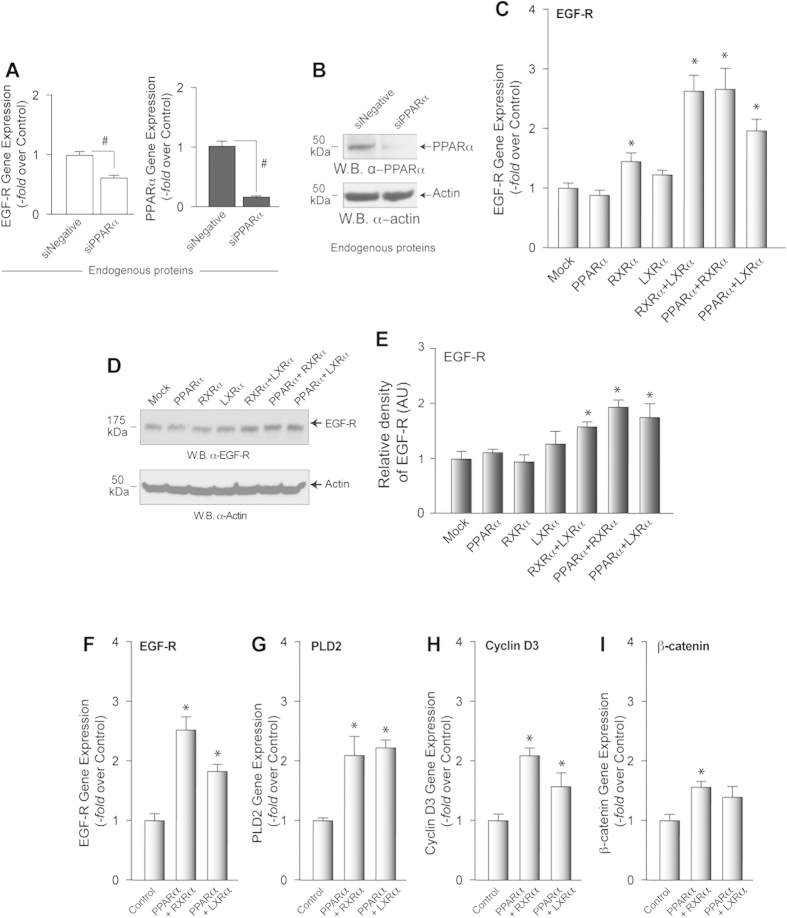
Effect of PPAR family of nuclear receptors on EGFR expression. (**A**) Silencing of PPARα (endogenous) with 200 nM siRNA for four days. Effect on EGFR expression and PPARα (control of silencing) by qPCR. (**B**) Western blot of endogenous protein silencing. (**C**) Effect of transcription of 3 different nuclear receptors (1 μg DNA) PPARα, RXRα or LXRα alone or in combination, on EGFR gene expression by qPCR. (**D**) Detection of EGFR protein mass by Western blot of cell lysates prepared from cell transfected with plasmids as in (**C**). (**E**) Densitometric analysis of three Western blots with similar experimental conditions as the one shown in (**D**). (**F–I)** Results of gene expression analyses for four target genes: EGFR (**F**), PLD2 (**G**), cyclin D3 (**H**) and β-catenin (**I**) with RNAs derived from cells overexpressing (1 μg DNA each) PPARα + RXRα or PPARα + LXRα. The blots presented in **B** have been cropped to depict the region around 50 kDa; and in (**D**), regions around 50 kDa and 175 kDa as indicated; all gels were run under the same experimental conditions. Experiments in this figure were performed in triplicate for at least 3 independent sets in total (n = 9). Results are mean +/- SEM and are expressed in terms of gene expression. The * symbols denote statistically significant (P < 0.05) ANOVA increases between samples and controls. The # symbols denote statistically significant (P < 0.05) ANOVA decreases between samples and controls.

PPAR family of nuclear receptors (NRs) uses free fatty acids as ligands. Moreover, PPAR regulates lipid metabolism and vice versa^38^. Our first question was to investigate if the PPAR family of transcription factors had any effect on expression of genes outside of lipid metabolism (for this, we chose EGFR as it is a target of PPARα modulation) and if lipids could still regulate such an effect. We first performed experiments modulating the expression of the NRs, PPARα, RXRα and LXRα, to ascertain what effect they had on EGFR expression. Figure 1A shows that silencing PPARα lowers the expression of EGFR indicating that this nuclear receptor mediates, at least in part, the regulation of EGFR expression. The figure also shows the control of silencing of PPAR. Figure 1B is a Western blot to demonstrate effective protein silencing. Since it is well established that the PPAR family of receptors function in heterodimers, we performed co-transfections with possible heterodimer NR partners of PPPARα (RXRα and LXRα) An increase in EGFR protein expression was observed with co-transfection of NRs (Fig. 1C–E). Overexpression of NRs is shown in Fig. 1F–I. The importance of dimerization has been shown in previous studies^25^,^26^,^27^,^28^. In order to know if this NR-mediated effect was EGFR-specific or more global, we tested the effect of NRs on other proteins. In addition to EGFR, PPARα heterodimers show positive effects on PLD2, cyclin D3 and β-catenin protein expression (Fig. 1F–I), indicating that the effect is not restricted to EGFR and in fact, influences several responses in the cell associated with an enhancement of gene expression.

### The combination PA+PPARα has a negative effect on EGFR expression

**Figure 2:**
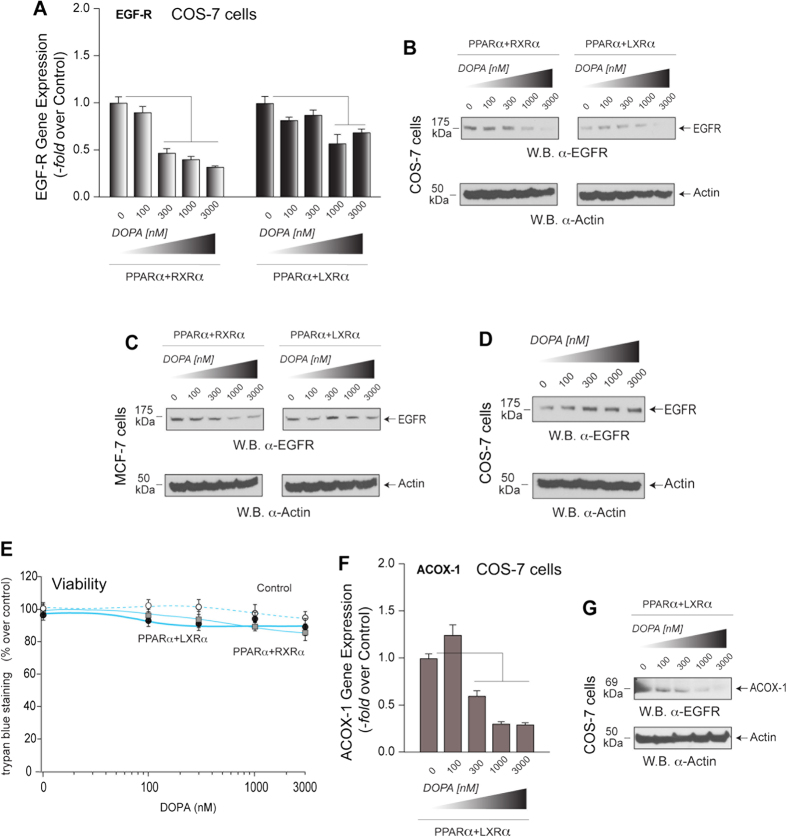
Effect of increasing concentrations of PA on EGFR gene or protein expression in the absence or presence of NRs heterodimers. (**A**) COS-7 cells overexpressing (1 μg DNA each) PPARα + RXRα or PPARα + LXRα were incubated with the indicated concentrations of 1,2,-dioleyl phosphatidic acid (DOPA), after which cells were used for RNA isolation, reversed transcribed to cDNA and analyzed by Q-PCR with EGFR primers and probe. (**B,C)** Western blot analyses of EGFR protein expression in COS-7 (**B**) or MCF-7 cells (**C**) overexpressing nuclear receptors in the presence of increasing concentrations of DOPA. (**D**) Effect of PA (alone, i.e., no NRs transfected) on EGFR protein expression using Western blot analysis. (**E**) Cell viability (by trypan blue) of PPARα + RXRα or PPARα + LXRα transfected cells treated with increasing concentrations of DOPA. (**F**,**G**) Effect of increasing concentrations of PA on EGFR and ACOX-1 gene or protein expression in the absence or presence of NRs heterodimers. COS-7 cells overexpressing (1 μg DNA each) PPARα + RXRα or PPARα + LXRα were incubated on the plates with the indicated concentrations of DOPA for 4 hours, after which cells were used for either RNA isolation, reverse transcription to cDNA and analysis by Q-PCR with EGFR or ACOX-1 primers and probes (**F**) or they were used to obtain cell lysates for Western blot analyses with anti-ACOX-1 antibodies (**G**). The blots presented have been cropped to depict the regions around 50 kDa and 175 kDa; and regions around 34 kDa, 50 kDa, 100 kDa and 105 kDa as indicated; all gels were run under the same experimental conditions. Experiments in this figure were performed in triplicate, and statistics and symbols are as indicated in the legend to Fig. 1.

As PPAR NRs use lipids as ligands, we wanted to look at the effect of PLD2’s lipase product, PA, on EGFR expression. Unexpectedly, we found that PA has a dose-dependent negative effect on PPARα-mediated EGFR expression (Fig. 2A), although the effect is more obvious in the case of PPARα + RXRα than in PPARα + LXRα (Fig. 2A). This negative effect on EGFR expression is also seen at the protein level in the two cell lines tested (COS-7 and MCF-7) (Fig. 2B,C). This negative effect is not due to PA per se, as Fig. 2D indicates that the effect of PA alone is not inhibitory (it is actually stimulatory) for EGFR protein expression. Figure 2E suggests that cell viability (by trypan blue) remains >90% even at high concentrations of PA. We next set out to answer if these effects have any specificity for the EGFR- or (aside from a small number of other genes) PPARα-mediated gene expression in general. We tested the effect of PA on PPARα-mediated gene expression of a very well-known target, acyl-CoA oxidase-1 (ACOX1). When COS-7 cells were transfected with both PPARα and LXRα (Fig. 2F), ACOX1 gene expression was repressed as a result of increasing PA concentration, which indicates that specificity of PA negatively affected PPAR-mediated global gene expression. This is also supported by western-blot analysis of similarly treated COS-7 cell lysates (Fig. 2G) that were probed with anti-ACOX1 antibodies (Fig. 2H). This could then be taken as a positive control, since ACOX-1, a known gene that is under regulation of PPARα, also affected by PA in a similar fashion as EGFR.

**Figure 3:**
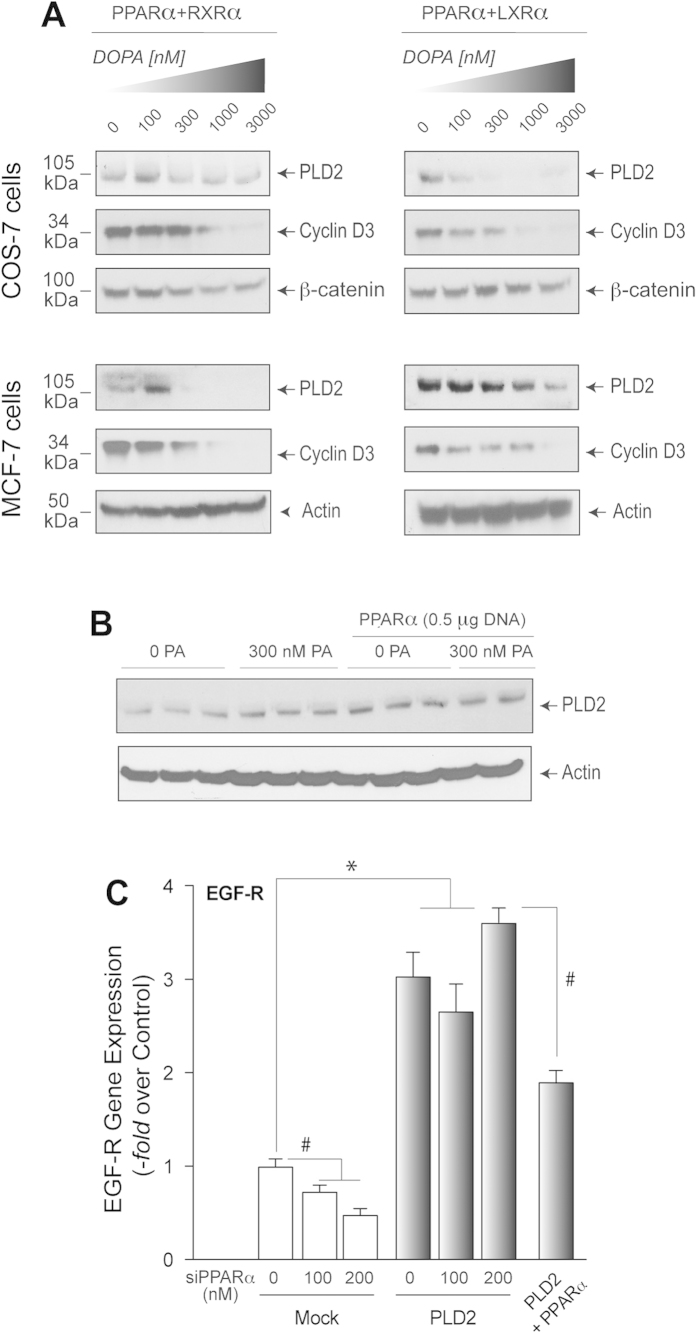
Effect of increasing PA concentration on PPARα-mediated protein expression of PLD2, cyclin D3 and β-catenin. (**A**) Western blot analysis of PLD2, cyclin D3 or β-catenin was performed with either COS-7 or MCF-7 cells that were subjected to the same conditions as in Fig. 2B,C. (**B**) PLD2 protein expression detected by Western blot analysis from cells treated as indicated, with either PA alone or in combination with NRs. (**C**) Effect of PLD2 transfection on EGFR gene expression in the absence or presence of silencing or overexpressing PPARα. The blots presented in panels A-B have been cropped to depict the regions around 50 kDa and 175 kDa; and panel E, regions around 34 kDa, 50 kDa, 100 kDa and 105 kDa as indicated; all gels were run under the same experimental conditions. Experiments in this figure were performed in triplicate, and statistics and symbols are as indicated in the legend to Fig. 1.

In addition to EGFR, PA’s negative effect on PPARα-mediated protein expression was also observed with PLD2, cyclin D3 and β-catenin, which are also growth and proliferation-promoting proteins^39^,^40^,^41^ (Fig. 3A). To investigate further the effect of PA on PLD2 itself, we subjected cells to the treatments indicated in Fig. 3B and found that PA or PPARα, separately, have a positive effect on PLD2 protein expression. However, in the presence of both PA and PPARα, expression of PLD2 is not augmented any further (rather, it is slightly diminished), which is in agreement with the results indicated earlier for EGFR. Lastly, Fig. 3C indicates that: *(a)* silencing PPARα reduced EGFR gene expression in agreement with the results in Fig. 1; *(b)* PLD2 enhances expression of EGFR, which is slightly elevated in PPARα-silenced cells (at 200 nM siRNA), and *(c)* PLD2 + PPARα significantly lowered EGFR gene expression level.

### Modulation of the transactivation activity of PPARα by PA

**Figure 4:**
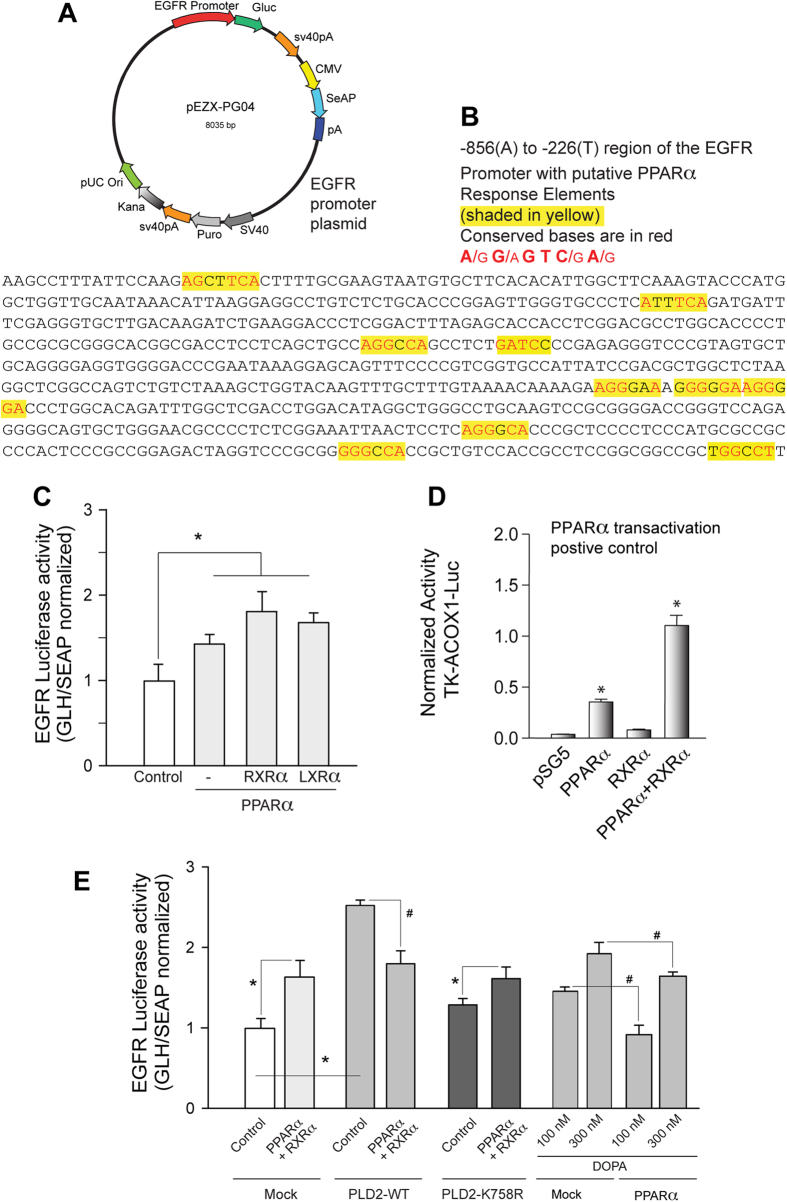
Transactivation activity of the EGFR promoter upon biding of nuclear receptors. (**A**) Schematic of EGFR promoter cloned into the pEZX-PG04 backbone. (**B**) EGFR promoter with putative PPAR response elements shaded in yellow and conserved bases of Response Element (RE) motifs shaded in red. (**C**) Luciferase assay of cells overexpressing EGFR promoter reporter plasmid (pEZX-PG04-EGF-R promoter) in the presence of co-transfected nuclear receptors. (**D**) Luciferase assay with TK-ACOX1-Luc, a positive control for trans-activation, to indicate validity of reagents and cells. (**E**) Effect of PLD2 (WT or lipase-inactive K758R mutant) and PA on the PPARα-mediated transactivation of EGFR promoter.

In order to ascertain the mechanism for the decrease in EGFR expression with the PPARα + PA (or PPARα + PLD2) combination, we cloned a fragment of the EGFR promoter into a reporter (luciferase) plasmid, the schematic of which is shown in Fig. 4A. Figure 4B shows several putative PPARα response elements in the EGFR promoter region spanning from -856(A) to -226(T). Figure 4C shows that the transfection of the reporter plasmid with PPARα and RXRα (or LXRα) resulted in increased luciferase activity, which in turn reflected an increase in EGFR promoter transactivation. Figure 4D is a positive control, where the positive effect of PPARα and PPARα + RXRα on the known target ACOX gene is observed.

To confirm the results we observed in Fig. 3B, we performed luciferase activity assays of EGFR promoter in combination with PLD2 and/or PPARα + RXRα. Results in Fig. 4E demonstrate that the individual effect of either PLD2 or PPARα + RXRα was positive on EGFR luciferase activity, while the effect was negated in the presence of PLD2 lipase inactive mutant K758R. We would expect to see a similar result in the presence of PLD2-specific inhibitors (one such example would be FIPI), or EGFR inhibitor, like mastoparan, could inhibit EGFR activity. Nakahata *et al*. showed that mastoparan was an inhibitor of phospholipase C (PLC)^42^, while Chahdi *et al*. showed that mastoparan functioned as a selective activator of PLD2^43^. Additionally, Chahdi *et al*. also showed in this same study that phosphatidylinositol 4,5-bisphosphate and oleate were competitive inhibitors of the mastoparan stimulation of PLD2^43^. However, in the presence of PPARα + RXRα, the PLD2-mediated effect was significantly lower. This trend was also observed when using PA instead of overexpressed PLD2 (Fig. 4E). Overall, the results from Figs 3 and 4 indicate that PLD2/PA in combination with PPARα repressed EGFR expression, while separately they exerted a positive effect.

### PA directly binds to PPARα as assessed by Circular Dichroism

**Figure 5:**
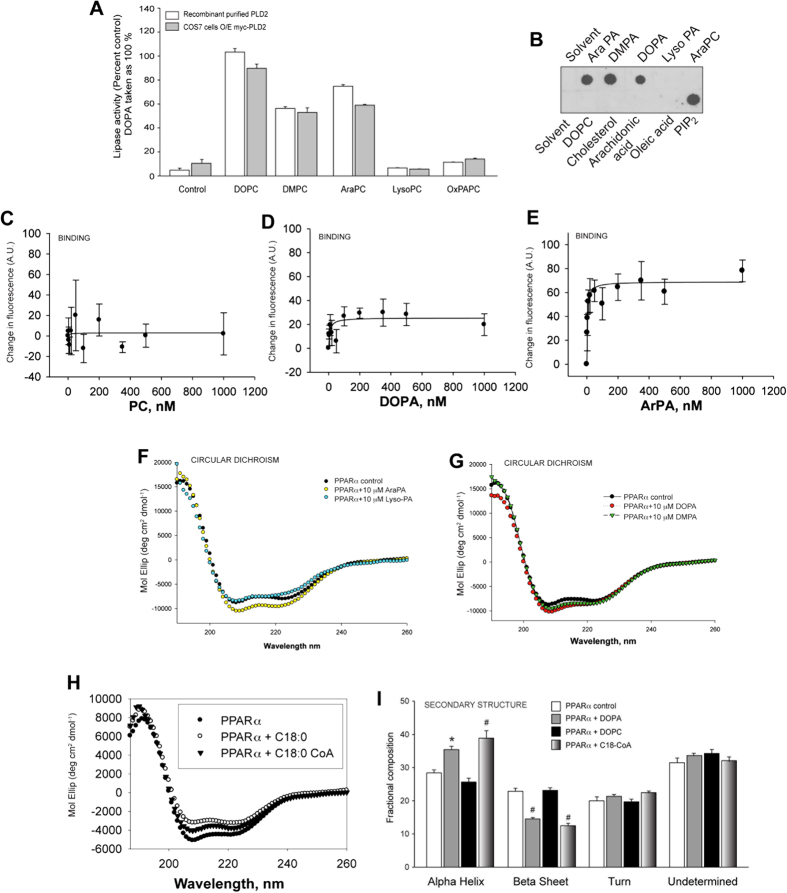
Binding and circular dichroism of PPARα in the presence of PA. (**A**) Phospholipase assay was performed using recombinant PLD2 and cells overexpressing PLD2 in the presence of several phospholipids: potential PLD substrates, 1,2-dioctanoyl-sn-glycero-3-phosphocholine (DOPC); 1,2-dimirystoyl-sn-glycero-3-phosphocholine (DMPC); 1,2-diarachidonoyl-sn-glycero-3-phosphocholine (AraPC); 1-oleoyl-2 -hydroxy-sn-glycero-3-phosphatidic acid (lyso-PC) and oxidized 1-palimitoyl, 2-arachidonoyl—sn-glycero-3-phosphocholine (OxPAPC). DOPC is considered the best substrate (positive control). (**B**) Protein-lipid overlay assays to PVDF membranes were performed with recombinant PPARα. PIP_2_ was used as a positive control for biding to PPARα and cholesterol was used as a negative control. (**C–E**) Protein-lipid binding by quenching of intrinsic aromatic amino acid fluorescence using the lipids: PC (**C**), DOPA (**D**) or AraPA (**E**) with recombinant PPARα *in vitro*. (**F,G**) Circular dichroism of PPARα upon binding to (**F**) AraPA (yellow circles) or lysoPA (blue circles), or (**G**) DOPA (red circles) or DMPA (green circles). (**H**) Positive control for Circular Dichroism; PPAR bound to C18:0-CoA, its strongest lignad. (**I**) Secondary structure analysis to ascertain the percentage of alpha helices, beta sheets, turns and undetermined structures in PPAR in the presence of PC or PA. C18:0-CoA was used as a positive control.

We hypothesized that PA could change the protein conformation of PPARα. This was also important considering that it has not been demonstrated to date whether or not PA can bind to and act as a ligand of PPARα. To investigate if PA, binds to PPAR, we first asked if other phosphatidylcholine substrates could be metabolized by PLD2 that could yield PA products. Figure 5A indicates that PLD lipase activity could produce PA from 4 different PC species: 1,2-dioleoyl-sn-glycero-3-phosphatidyl choline (DOPC), 1,2-dimyristoyl-sn-glycero-3-phosphatidyl choline (DMPC) and 1,2-diarachidonoyl-sn-glycero-3-phosphatidyl choline (AraPC), 1-oleoyl-2 -hydroxy-sn-glycero-3-phosphatidic acid (lyso-PC) and oxidized 1-palimitoyl, 2-arachidonoyl-sn-glycero-3-phosphocholine (OxPAPC). The data indicates that DOPC was the preferred substrate and DOPA was the preferred product (that is the one we have been using throughout this study and the one that we tested next for its efficiency to bind to PPARα).

Next, binding of lipids to PPARα was investigated using two different techniques: protein-lipid overlay assays to PVDF membranes and quenching of intrinsic aromatic amino acid fluorescence. For the first technique, Fig. 5B shows that DOPA binds to PPARα, which is in agreement with the binding of cyclic phosphatidic acid (cyclic-PA) to another member of the PPAR familiy, PPARγ^44^,^45^. Binding assays were also performed by quenching the intrinsic aromatic amino acid fluorescence and Fig. 5C–E confirmed binding of PPARα to DOPA and AraPA but not to PC.

### Changes in PPARα secondary structure and effect on recruitment of NR heterodimers

To investigate if there were any structural changes that could occur upon binding of PA to PA-PPARα, we used Circular Dichroism (CD). As seen in Fig. 5F,G the binding of PA to PPARα changed the molar elliptical spectra of PPARα, which did not occur with lyso-PA (which is not a product of the PLD2 reaction). Shown also is a positive control for Circular Dichroism with the same PPARα protein preparation bound to its strongest ligand, C18-CoA (Fig. 5H)^25^,^35^. Figure 5F,G indicate that upon binding of PA to PPARα, a significant structural change occurred. Figure 5I shows that this change manifested as a significant increase of alpha helices at the expense of beta sheets, which decreased in the nuclear receptor’s secondary structure. In the presence of DOPA, both α-helical and β-sheet content changes were significantly altered comparable to those of the known positive control C18-CoA (Fig. 5I)^25^,^35^.

**Figure 6:**
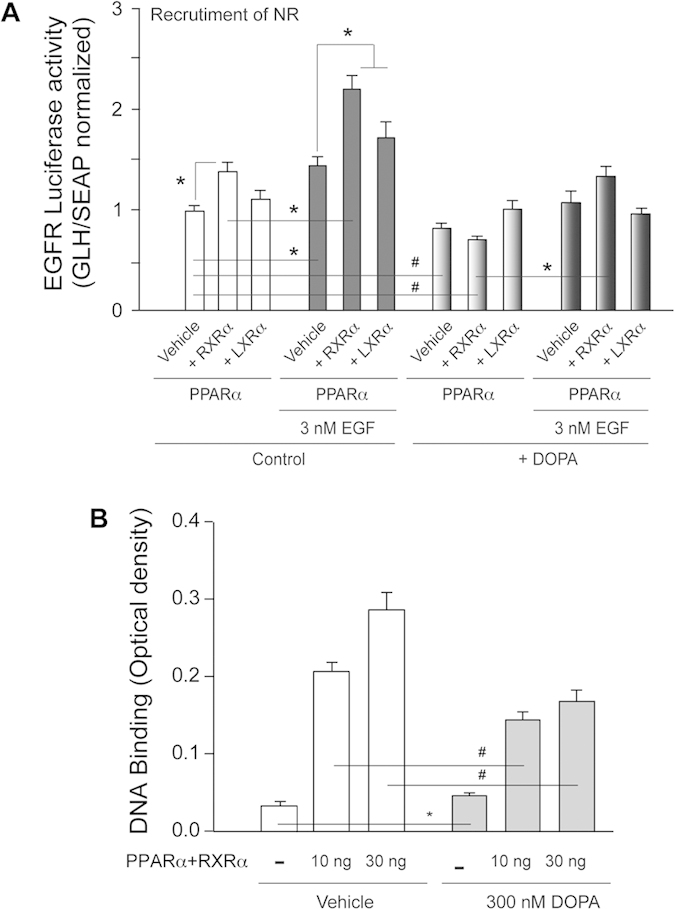
Effect of EGF on PPARα recruitment and binding to the EGR promoter in the absence or presence of PA. (**A**) Effect of EGF on PPARα/RXRα/LXRα mediated transactivation of EGFR promoter. Pre-incubation with 3 nM EGF enhanced the NRs-mediated positive effect on EGFR promoter activity, especially when heterodimers are present. The presence of 300 nM PA has an overall negative effect irrespective of the absence or presence of EGF. (**B**) *In vitro* binding of dsDNA with a putative RE binding sequence to PPARα (derived from Fig. 4B). *In vitro* assay was performed as indicated in Materials & Method section. Control had 30 ng/well BSA instead of PPARα. PA was used at the concentration of 300 nM.

Results in Fig. 4E demonstrated that while separately PLD2 and PPARα have a positive effect on the receptor expression, in combination EGFR promoter activity was lowered. Since it is known that EGF is the positive effector of EGFR expression^46^, we wanted to determine the effect of EGF on EGFR promoter activity. Addition of 3 nM EGF enhanced PPARα-mediated EGFR promoter activity (Fig. 6A). However, the presence of PA (300 nM for 4 h) had a negative effect on NR-mediated EGFR promoter activity regardless of the presence of EGF (Fig. 6B). Overall these results suggest that recruitment of other NRs to form heterodimers with PPARα was compromised and pointed to an explanation as to why PA decreased PPARα-mediated EGFR promoter activity.

### PA alters the binding of PPARα to the EGFR promoter

To further understand the mechanism behind the involvement of PA and PPARα in modulating EGFR promoter activity, we investigated if PPAR could bind to the EGFR promoter in vitor and, if so, if this binding could be altered in the presence of PA. The results of EMEA technique (as described in the methods section) are shown in Fig. 6B. PPARα shows a dose-dependent increase in DNA binding (white bars), indicating that PPAR indeed binds to the indicated region of the promoter *in vitro*, whereas PA at 300 nM showed a negative effect on PPARα’s ability to bind to DNA. Thus, we concluded that PPARα binds to the EGFR promoter in the region TTCCAAG**AGCTTCA**CTTTTGCGAAGTAATGTGCTTCACACATTGGCT [-840(A) and -834(A)] and enhance EGFR expression, which is compromised in the presence of 300 nM PA. All this is in agreement with data in Fig. 4E and Fig. 6A.

### PA + PPARα in aggressive breast cancer cells

**Figure 7:**
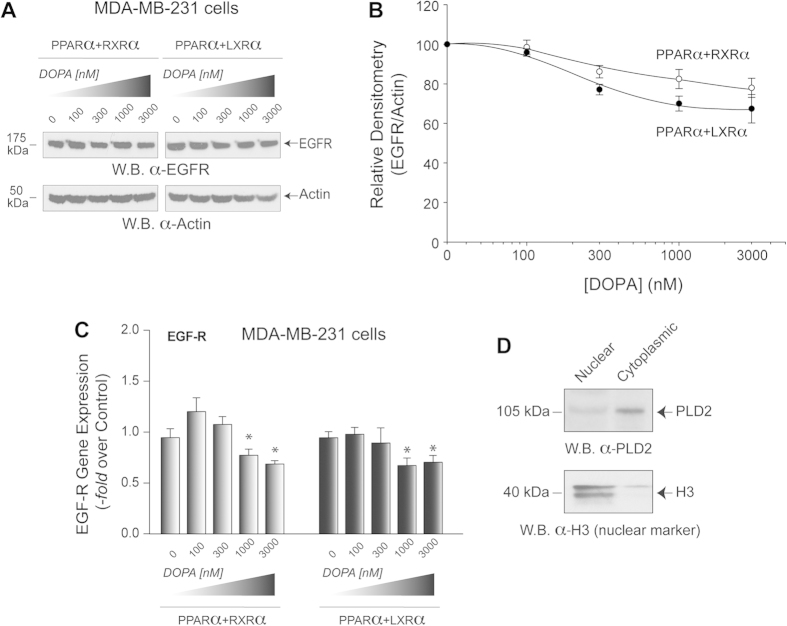
Effect of PA on MDA-MB-231 cells overexpressing NR heterodimers. (**A**) Basal breast cancer cells MDA-MB-231 were transfected with either PPARα + RXRα or PPARα + LXRα plasmids for 2 days. Cells that were incubated with the indicated concentrations of DOPA were analyzed by Western blots for EGFR protein expression. (**B**) Graphical quantification of the EGFR densities shown in (**A**) relative to actin staining. (**C**) In parallel, cells treated similarly, were used for RNA extraction and Q-PCR to quantify EGFR gene expression. Experiments were performed in triplicate, and statistics and symbols are as indicated in the legend of Fig. 1. (**D**) Western blot analysis of cells after subcellular fractionation and ultracentrifugation, indicating that PLD2 is present mainly in the cytoplasm, but also in the nucleus. The histone-3 (H3) is a nuclear marker. Experiments in this figure were performed in triplicate, and statistics and symbols are as indicated in the legend to Fig. 1.

We next wanted to test the non-additive effect of the PA + NRs combination in EGFR expression in the cancer cell line MDA-MB-231 (which is more aggressive than the MCF-7 cancer cell line tested in Fig. 2), where both PLD2 and EGFR are abundantly expressed. Also, PPARα levels, as well as its enzymatic activity, regulate the proliferative ability of these cells^47^. The results represented in Fig. 7A–C suggest that PA still negatively affected PPAR-mediated EGFR expression but to a lesser extent when compared to the other two cell lines (COS-7 and MCF-7) tested previously. The negative effect of PA on PPAR-mediated EGFR expression was first observed at >1000 nM PA (Fig. 7A–C), unlike in the other cell lines where 300 nM PA significantly exerted a negative effect (Fig. 2). This might be due to the fact that MDA-MB-231 cells express EGFR to a greater extent than COS-7 or MCF-7 cells, indicating other additional signaling pathways which might be involved in promoting EGFR expression in MDA-MB-231 cells compared to COS-7 or MCF-7 cells. Lastly, Fig. 7D indicates that PLD2 is maninly located in the cytoplasm (associated to membranes) but there is a fraction (~20%) that localizes in the nucleus. Either of these two pools, but particularly the latter, could contribute to PA to exert the reported effects in this study.

## Discussion

EGFR promoter activity reflects gene and protein expression of EGFR and its kinase activity. We report here for the first time that PLD2 via its lipase product PA affects EGFR expression directly, and we also demonstrate the specific molecular mechanisms involved. Results in Figs 2, 3, 4 showed that either the presence of PLD2 or PA, along with PPARα + RXRα, lowered EGFR promoter activity. We implicate the regulation of EGFR promoter activity by nuclear receptors PPARα-RXRα heterodimers and report here for the first time that PA binds to PPARα causing a change in this protein’s secondary conformation. The mechanism proposed in the present study mainly considered breast cancer cells, such as MDA-MB-231 cells in which PLD2 was overexpressed. Zhang *et al*. demonstrated that cellular PA levels increased in cells overexpressing PLD2 using the PA sensor plasmid^38^. Unexpectedly, PLD2 generated PA was responsible for a reduction in PPAR-mediated EGFR promoter activity and PA together with the nuclear receptors caused a transrepression of the EGFR expression.

In addition, we identified putative PPARα response elements upstream of the start site of the EGFR promoter. Our experiments revealed that overexpression of NRs exert a positive effect on multiple genes including EGFR, PLD2, cyclin D3 and β-catenin. An interesting phenomenon that we observed is the negative effect of PA on NRs-mediated gene expression (Fig. 2). We have demonstrated for the first time that PA binds to PPARα (Fig. 5). Also, through luciferase assays that we performed with the EGFR promoter, we revealed that transfection of PLD2 along with the NR provided a lower luciferase activity, which is in agreement with earlier findings in this study. Addition of EGF to cells prior to harvesting could not reverse the negative effect of the combination of PLD2 + NR. The combination of PA and PPARα once again decreased EGFR luciferase activity (Fig. 6A). PPAR transcription factors are known for their transactivation, as well as their transrepression capabilities. It is the latter function of the PPARs that is involved in suppression of pro-inflammatory mediators^48^.

Several scenarios might explain why PA or PPARα alone increase gene expression, while their combination is not additive or synergistic but rather, detracts from gene expression. First, we could speculate that binding of PA to PPARα interferes with PPARα’s binding to the EGFR promoter. *In vitro* DNA binding experiments with PPRE elements of the EGFR promoter and recombinant PPARα reveals that PPARα’s ability to bind DNA is significantly reduced in the presence of PA (Fig. 6B). The results of this report also indicate that PA + PPARα binding to the promoter failed to recruit the necessary NRs RXRα or LXRα to form a fully functional heterodimers.

A third scenario exists that in the PA + PPARα combination, co-suppressors might be recruited to the EGFR promoter resulting in reduced EGFR expression. This was demonstrated for SMRT 44. Lastly, another possibility could be that the cloned reporter could be missing other positive response elements. Ligand-(PA)-dependent transrepression of PPARα cannot be ruled out, where the ligand itself induced NR-mediated repression of the target gene. Transrepression by PPARs can occur many ways. PPARs directly interact with transcription factors, including AP-1, and interfere with the latter’s DNA binding ability, thus preventing the transcription of the target genes. Alternately, PPARs are also involved in recruiting or preventing the degradation of co-repressors and thus inhibit transcription of certain genes^49^.

Results from Figs 1, 2, 3, 4, 5, 6 show PA-mediated PPAR suppression of gene expression in COS-7 and MCF-7 cells and, as such, the significance is that the combination PA + PPARα is a new way to interfere with the expression of EGFR. The effect of this phenomenon on MBA-MB-231 cells slightly differs in that 1000 nM PA rather than 300 nM PA exerts the negative effect on PPARα-mediated EGFR gene expression (Fig. 7A,B). This might be due to the abundant levels of EGFR that the MDA-MB-231 cells express as a result of being a more metastatically aggressive cancer cell line.

**Figure 8:**
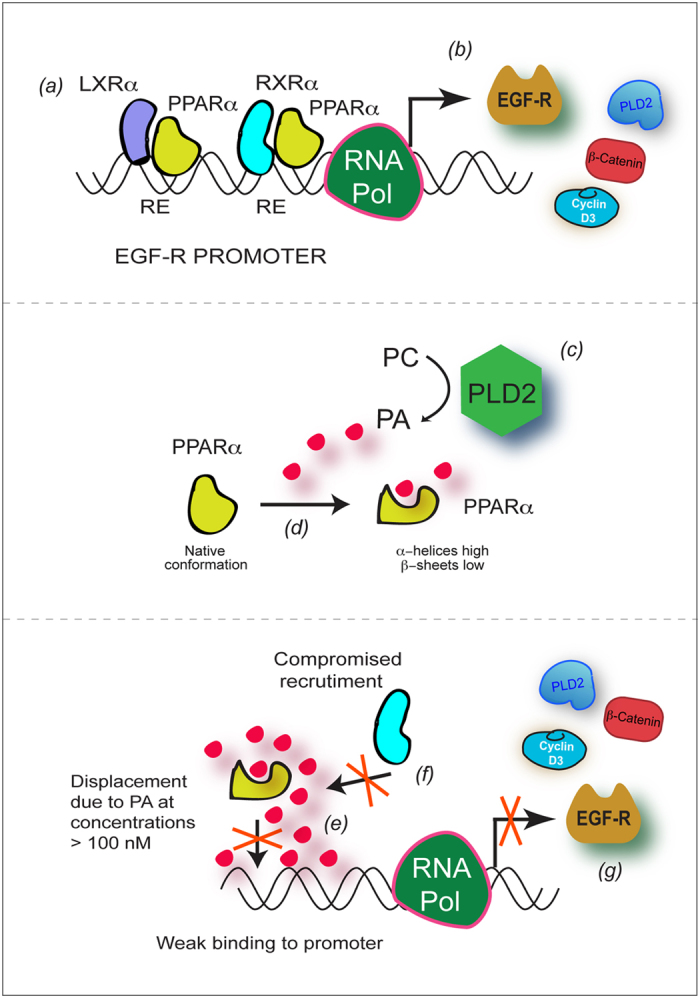
Model on how PLD/PA + NRs could repress EGFR expression. In the absence of PLD/PA (***a***), PPARα-LXRα or PPARα-RXRα heterodimers exert a positive effect on EGFR promoter activity (***b***), as well as in cyclin D3, β-catenin and PLD2 genes. When PLD2-generated PA (***c***) binds to PPARα it causes conformational changes in PPARα (***d***) that increase the α-helices content at the expense of the β-sheets content. Either this conformational change or the presence of PA at concentrations >300 nM, diminish the ability of PPARα to bind to the EGFR promoter (***e***) and/or the recruitment of RXRα to form functional dimers (***f***) leading to a repression of EGFR expression (***g***).

We propose a model in which we explain the regulation of gene expression by PPARα. In the absence of PLD/PA, PPAR binds to its response elements on the target gene promoters and exerts a positive effect (Fig. 8, top panels). When PA binds to PPARα, this leads to a conformational change in the secondary structure of the protein (increase in α-helices and decrease in β-sheets) that is sufficient to either prevent it’s own binding to the promoter or the new conformation avoids binding to the heterodimeric NR partner. Alternatively, ligand-dependent transrepression might be occurring at this point.

As known, binding of fatty acid ligands causes a conformational change in PPARα in a way that allows for PPARα to release its co-repressors and bind to its co-activators (e.g., ASC complex, CBP-SRC-HAT complex, or the TRAP-Mediator complex), resulting in the initiation of transcription of target genes.PPARγ binding to CPA results in repression^44^,^45^,^50^,^51^ with which our results are in agreement. Data in the present study indicate that conformational changes are indeed present (increase in the α-helices content at the expense of the β-sheets content). However, the functional result is different from what is stated above. Either this conformational change or the presence of PA at concentrations >300 nM diminished the ability of PPARα to bind to the EGFR promoter and/or the recruitment of RXRα to form functional dimers leading to repression of EGFR expression. We cannot rule out that PA could be metabolically converted to lyso-PA after incubation in cells and that part of this lyso-PA could mediate some of the responses indicated in this study. However, the presence of PA and enzymatically active PLD2 in nuclear membrane and nucleus validates the reported effects of PA.

Overall, results from the present study indicate that PPARα can act as both a transactivator (when acting alone) or as a transrepressor (when acting through PA) of EGFR, PLD2, cyclin D3 or β-catenin. We believe PA activates PPARα to be a transrepressor. In context with this thought, it has been shown that PPARγ and LXRα are involved in transrepression in a gene- and signal-dependent fashion^52^. Moreover, natural ligands of LXRs can determine LXR-mediated gene activation-repression, which might be the case for PPARα also. As the current study was performed in breast cancer cell lines, it suggests a key role for PLD2 in maintaining the levels of EGFR and could be used as a target for modulating its expression.

## Additional Information

**How to cite this article**: Mahankali, M. *et al.* Phosphatidic Acid (PA) can Displace PPARα/LXRα Binding to the EGFR Promoter Causing its Transrepression in Luminal Cancer Cells. *Sci. Rep.* **5**, 15379; doi: 10.1038/srep15379 (2015).
